# A Positive Causal Influence of IL-18 Levels on the Risk of T2DM: A Mendelian Randomization Study

**DOI:** 10.3389/fgene.2019.00295

**Published:** 2019-04-05

**Authors:** He Zhuang, Junwei Han, Liang Cheng, Shu-Lin Liu

**Affiliations:** ^1^Systemomics Center, College of Pharmacy, and Genomics Research Center (State-Province Key Laboratories of Biomedicine-Pharmaceutics of China), Harbin Medical University, Harbin, China; ^2^College of Bioinformatics Science and Technology, Harbin Medical University, Harbin, China; ^3^Department of Microbiology, Immunology and Infectious Diseases, University of Calgary, Calgary, AB, Canada

**Keywords:** interleukin-18 levels, type 2 diabetes mellitus, casual effect, Mendelian randomization, genome-wide association studies

## Abstract

A large number of clinical studies have shown that interleukin-18 (IL-18) plasma levels are positively correlated with the pathogenesis and development of type 2 diabetes mellitus (T2DM), but it remains unclear whether IL-18 causes T2DM, primarily due to the influence of reverse causality and residual confounding factors. Genome-wide association studies have led to the discovery of numerous common variants associated with IL-18 and T2DM and opened unprecedented opportunities for investigating possible associations between genetic traits and diseases. In this study, we employed a two-sample Mendelian randomization (MR) method to analyze the causal relationships between IL-18 plasma levels and T2DM using IL18-related SNPs as genetic instrumental variables (IVs). We first selected eight SNPs that were significantly associated with IL-18 but independent of T2DM. We then used these SNPs as IVs to evaluate their effects on T2DM using the inverse-variance weighted (IVW) method. Finally, we conducted sensitivity analysis and MR-Egger regression analysis to evaluate the heterogeneity and pleiotropic effects of each variant. The results based on the IVW method demonstrate that high IL-18 plasma levels significantly increase the risk of T2DM, and no heterogeneity or pleiotropic effects appeared after the sensitivity and MR-Egger analyses.

## Introduction

Type 2 diabetes mellitus (T2DM) is a complex metabolic disease and accounts for more than 90% of diabetic cases. Its pathogenesis involves both genetic predisposition and unhealthy living habits (Zheng et al., 2018). The disease occurs mostly after the age of 35–40 years, providing potential time windows for proactive strategies toward effective prevention (Palermo et al., 2014;Zheng et al., 2018).

Among the known risk factors, inflammation has been identified as a potential cause of T2DM as well as other obesity-associated diseases, such as atherosclerosis and fatty liver (Kohlgruber and Lynch, 2015; Zou et al., 2018). Inflammations interfere with glucose metabolism in adipocytes, hepatocytes, and muscle cells and also affect insulin production or signaling (Kohlgruber and Lynch, 2015). The IL-1 cytokine family, a major class of immunoregulatory agents, plays important roles in endocrinal processes and the regulation of responses to inflammatory stress, especially in T2DM (Banerjee and Saxena, 2012). For example, human pancreatic cells produce more IL-1β under higher glucose concentrations, which in turn may lead to impaired insulin secretion, decreased cell proliferation, and, eventually, β-cell death (Poitout and Robertson, 2002; Rhodes, 2005). In contrast, IL-1Ra, another member of the IL-1 family, can protect cultured human islets from high glucose-induced IL-1β-mediated β-cell apoptosis (Maedler et al., 2001). Obviously, members of the IL-1 family, e.g., IL1-Ra and IL-1β, maintain a dynamic balance to influence β-cell function and glycemic regulation in T2DM development (Larsen et al., 2007, 2009). Recently, interleukin-18 (IL-18), an IL-1 family member, has been reported to be involved in T2DM and play a role in regulating innate and adaptive immune responses (Matsui et al., 1997; Wawrocki et al., 2016). Immediately after this report, a nested case-control study based on the Nurses’ Health Study showed high IL-18 levels are associated with a higher risk of T2DM (Hivert et al., 2009). In another study, IL-18 levels were measured in serum samples from 130 coronary artery disease (CAD) patients. The study included 43 T2DM patients and 31 healthy controls and also revealed that T2DM patients tend to have higher IL-18 serum levels (Suchanek et al., 2005). These results are consistent with previous clinical findings that increased IL-18 serum levels serve as a marker of insulin resistance in both T2DM patients and non-diabetic people (Fischer et al., 2005). However, due to the interference of multiple confounding factors and the “reverse causal effect” in observational studies, it remains unclear whether high levels of IL-18 trigger the onset of T2DM and cause or push the development of the disease as a main confounding factor, an issue that calls for systematic investigations for the development of effective preventive or therapeutic strategies, e.g., by Mendelian randomization (MR) studies (Noyce et al., 2017; Schuetz and Wahl, 2017).

**FIGURE 1:**
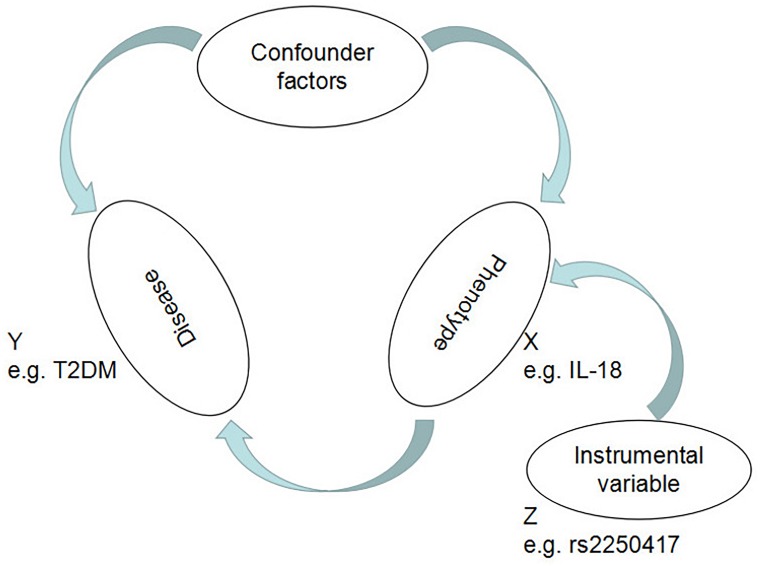
Mendelian randomization analysis utilizing genetic variants as instrumental variables for estimating the effect of IL-18 plasma levels on T2DM.

Mendelian randomization, greatly facilitated by the development of genome-wide association studies (GWASs), is a method for establishing causal effects between genetic traits and diseases by building instrumental variables (IVs) based on the information about single nucleotide polymorphisms (SNPs), i.e., phenotype-associated genetic variants (Visscher et al., 2012; Hayes, 2013; De et al., 2014; Huang, 2015; Li et al., 2015; Sekula et al., 2016; Zheng et al., 2017; Cheng et al., 2018d, 2019; Guo et al., 2018). For MR analysis, all IVs have to be independent of one another and robustly associated with the phenotype (e.g., high IL-18 levels) but not with the disease (e.g., T2DM) (Figure 1), ensuring that the only way for the IVs to influence the disease is through the phenotype, with maximum avoidance of any possible residual confounding factors. Based on Mendel’s second law, i.e., the principle of random distribution of gametes in offspring (Castle, 1903), IV analysis can avoid reverse causality.

In this study, we verified the assumption that T2DM is caused by high IL-18 levels. Next, we estimated the causal effect of IL-18 levels on T2DM by the MR method.

## Materials and Methods

### Strategic Design of Data Processing and Analysis

**FIGURE 2:**
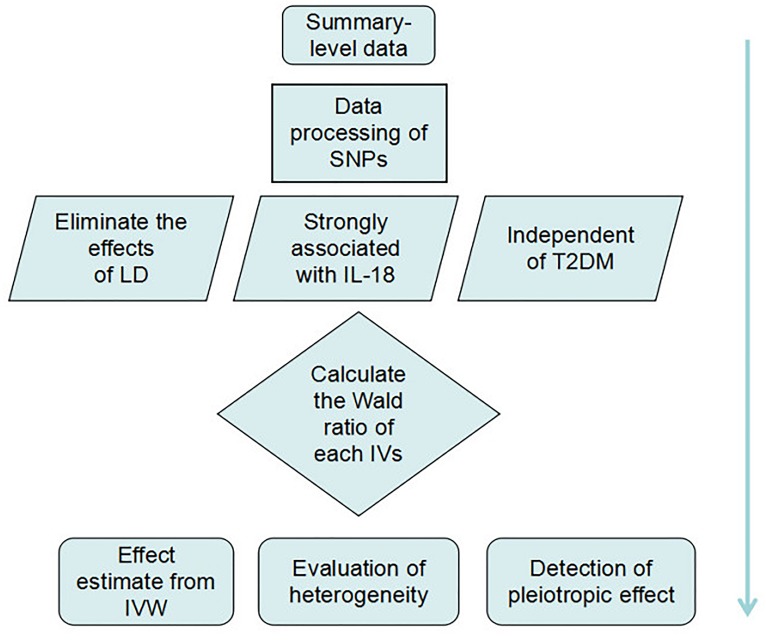
Strategic design of data processing and analysis.

We extracted summary-level data from GWAS datasets and processed the data by removing the SNPs not suitable for establishing IVs. We then calculated the Wald ratio of each IV, and we used the inverse-variance weighted (IVW) method to predict the causal effects of high IL-18 serum levels on T2DM. Upon completing the MR analysis, we evaluated the heterogeneity and pleiotropic effects of each variant, using the sensitivity analysis and the MR-Egger method, respectively (Figure 2).

### Summary-Level Data Extraction for Associations Between Genetic Variants and IL-18

The SNP information required to construct the IVs was extracted from a meta-analysis study done in 2013 by Walston et al. This team identified 18 top significant SNPs associated with plasma IL-18 levels (*P* < 5 × 10^−8^), using the GWAS data from the Cardiovascular Health Study (CHS) and a prospective population-based cohort study called InCHIANTI (Matteini et al., 2014). The “haplo.glm” function, implemented by the original author in the “haplo.stats” R package, was used to calculate the beta coefficient (β), the standard error (SE), and the threshold of the *P-value* for each haplotype relative to the most common reference haplotype (Matteini et al., 2014). Participants in this study included 3233 individuals over the age of 65 from the CHS cohort, and another group of 1210 participants aged 65–102 years from the InCHIANTI cohort, all being Caucasian (Fried et al., 1991; Ferrucci et al., 2000). The related SNP serial numbers, allele frequencies, effect alleles (EAs), beta coefficients, and SEs were obtained from the meta-analysis results by combining the two cohorts.

### Summary-Level Data Extraction for Associations Between Genetic Variants and T2DM

The GWAS data used for this study were obtained from the *trans-*ethnic T2D GWAS meta-analysis for calculating the subsequent Wald ratio. In total, 26,488 T2DM cases and 83,964 controls were used in the study, and 2,915,012 genetic variants were identified, which have been published by the Diabetes Genetics Replication and Meta-analysis (DIAGRAM) consortium^1^. The odds ratio (OR), SE, and *P-value* of T2DM per allele were extracted. The *P-value* established for screening genotypes (*P* < 5 × 10^−8^) independent of type 2 diabetes is specifically referenced to a number of similar studies; the most authoritative of which is “Estimating the causal influence of body mass index on risk of Parkinson disease: A Mendelian randomization study,” which was published in PLOS Medicine in 2017.

### Data Processing

Due to the potential linkage disequilibrium (LD), the IVs were chosen independent of each other to avoid over-precise estimates in subsequent analysis caused by genetic pleiotropy. According to the application principles of MR analysis, the study is based on Mendel’s second law of inheritance: the separation and combination of gene pairs controlling different traits do not interfere with each other; in the formation of gametes, the paired genes are separated from each other, and genes that determine different traits are randomly distributed between two gametes. When two genes are not completely independent, they will show a certain degree of linkage; this situation is called LD, and it greatly affects the exclusiveness of the variable tool to phenotypic inheritance, leading to subsequent calculation bias, generally called “over-precise estimates” (Noyce et al., 2017). Although rudimentary selection has been applied by Walston et al. (Matteini et al., 2014), the processed SNPs were verified again using an LD web tool^2^ to remove the interfering SNPs (r2 threshold = 0.1 or within 500 kb physical distance) (Baird, 2015; Noyce et al., 2017). Next, the T2DM-related SNPs (*P* < 0.05) were removed to meet the conditions for the MR analysis, making the IL-18-associated variants independent of the disease.

### MR Method

Mendelian randomization is a method applied by pooling Wald ratios of the IVs to verify the causal relationship between exposures and diseases (Emdin et al., 2017). The Wald ratio of each IV was calculated first. As shown in Figure 2, we assumed *X, Y*, and *Z* to be IL-18, T2DM, and IVs, respectively, and the Wald ratio (*β_XY_*) of IL-18 to T2DM through a specified variant can be calculated as follows:

$${\text{β}}_{\text{XY}}={\text{β}}_{\text{ZY}}/{\text{β}}_{\text{ZX}},$$

where β_ZY_ represents the per-allele *log(OR)* of T2DM from summary-level data of Morris et al. (Morris et al., 2012), and β_ZX_ is the per-allele *log(OR)* of IL-18 from summary-level data of Walston et al. (Matteini et al., 2014). The SE of the IL-18–T2DM association of each Wald ratio can be defined as follows:

$${\mathrm{SE}}_{\text{XY}}={\mathrm{SE}}_{\text{ZY}}/{\mathrm{SE}}_{\text{ZX}},$$

where *SE*_ZY_ and *SE*_ZX_ represent the SE of the variant–T2DM and variant–IL-18 associations from corresponding summary-level data, respectively. Subsequently, 95% confidence intervals (CIs) were calculated from the SE of each Wald ratio. Then, these data were pooled to estimate a weighted average of the causal effect by the IVW method. This method is one of the most commonly used methods for meta-analysis of fixed effects models. It summarizes effect sizes from numerous independent studies by calculating the weighted mean of the influence sizes, taking the inverse variance of individual studies as weights. The meta-analysis model for the point estimate is on the basis of the heterogeneity of the pooled data. The fixed effect model is applied for the case of no significant heterogeneity, while the random-effect model is used for others (Boucher, 2012; Lee et al., 2016).

In order to assess the genetic heterogeneity of summarized data, Cochran’s *Q*-test and the I^2^ statistic were applied. Cochran’s *Q*-test applies a χ^2^ distribution with (*k*-1) degrees of freedom, where *k* is the number of variants for analysis; *I*^2^ = [Q - (*k* - 1)]/*Q* × 100% ranges from 0 to 100%. *P* < 0.01 and *I*^2^ > 50% are defined as significant heterogeneity (Zhang et al., 2015).

### Leave-One-Out Method for Sensitivity Analysis

The sensitivity analysis was conducted to detect the heterogeneity of each variant, and the IVW method was carried out for each set of variants without a “missing SNP” to get the point estimates from IL-18 on T2DM (Noyce et al., 2017). Then, we checked the fluctuation of the results before and after removing the “missing SNP,” which reflects the sensitivity of each IV (Zheng, 2017).

### MR-Egger Method

MR-Egger regression analysis was applied here to ensure that violations in the analysis would not bias the estimates of the directional causal association (Bowden et al., 2015). The MR-Egger regression analysis was originally derived from the Egger regression method, which is mainly used to detect research bias in meta-analysis and systematic bias caused by pleiotropy. The estimated value of the intercept from MR-Egger regression can be interpreted as an estimate of the average pleiotropic effect across the genetic variants. Estimates of the average pleiotropic effect of genetic variants can be reflected in the intercept estimates in MR-Egger regression. A non-zero intercept is indicative of overall directional pleiotropy, and the slope coefficient provides a bias estimate of the causal effect (Bowden et al., 2015). All above statistical analyses were conducted in R 3.4.3 using the R package of meta-analysis 1 and MR^3^.

## Results

### IV SNPs

A selection of eight SNPs to construct IVs (rs2250417, rs2300702, rs2268797, rs6748621, rs7577696, rs6760105, rs212745, and rs212713) satisfied all conditions, including strong associations with IL-18 phenotypes (*P* < 5 × 10^−8^, β ≠ 0) and no association with T2DM (*P* > 0.05) or LD effect (Table 1).

**Table 1:** Associations of genetic variants with IL-18 and T2DM.

SNP	Chr	Gene	BP	IL-18	IL-18	IL-18 *P*	T2DM β	T2DM	T2DM
							
				β	SE			SE	*P*
rs2250417	11	BCO2	111590526	0.1	0.01	1.9 × 10^−32^	0.00995	0.0102	0.71
rs2300702	2	SRD5A2	316411522	0,07	0.01	1.6 × 10^−17^	0.00995	0.0153	0.29
rs2268797	2	SRD5A2	31637256	0.07	0.01	2.8 × 10^−17^	0.00995	0.0153	0.26
rs6748621	2	DPY30	32115705	0.08	0.01	1.1 × 10^−16^	0.00995	0.0102	0.68
rs7577696	2	DPY30	32132286	0.08	0.01	2.7 × 10^−19^	0.00995	0.0102	0.6
rs6760105	2	SPAST	32160890	0.06	0.01	3.6 × 10^−16^	0.00995	0.0102	0.61
rs212745	2	SLC30A6	32266336	0.07	0.01	2.1 × 10^−15^	0.00995	0.0102	0.64
rs212713	2	NLRC4	32311041	0.06	0.01	1.5 × 10^−10^	0.00995	0.0102	0.61

### The Causality Influence From BMI on the Risk of T2DM

**FIGURE 3:**
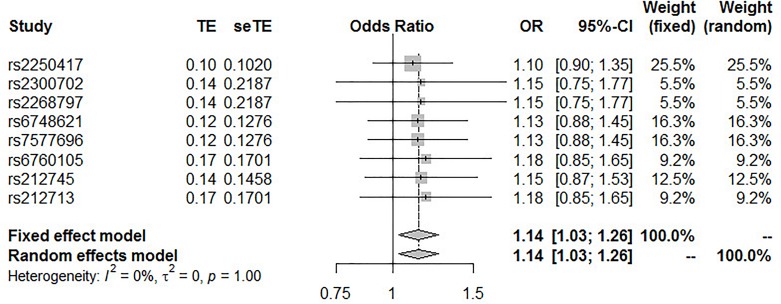
Forest plot revealing Wald ratios and 95% CIs from IL-18-associated SNPs.

The pooled results from the IVW method with eight SNPs suggest that high IL-18 plasma concentrations significantly increase the risk of T2DM. No heterogeneity was found between variants of the summary data (*P* = 1.0 and *I*^2^ = 0%; Figure 3); the fixed-effect model was applied for the meta-analysis, and the OR of T2DM per SD higher IL-18 plasma level was 1.14 (95% CI 1.03 – 1.26, *P* = 0.0117; Figure 3).

### Sensitivity Evaluation

**FIGURE 4:**
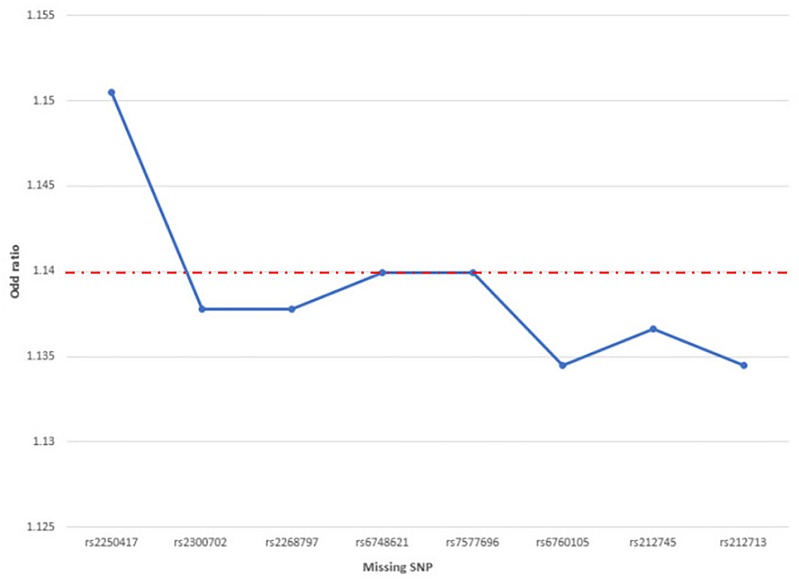
Scatter plot of the ORs from IL-18-associated SNPs in the “leave-one-out” analysis. The red baseline shows the results without missing any SNP; the blue dots denote the results after removing one SNP.

The ORs obtained after removing the “missing SNP” all exceeded 1, ranging from 1.1345 to 1.1505, with small fluctuations from -0.005 [(1.1345–1.14)/1.14] to 0.009 [(1.1505–1.14)/1.14]. This means that the causality effects we obtained from MR were supported by most of the individual SNPs, demonstrating that no single SNP dominated the IVW point estimate, and there was no heterogeneity in the variants (Figure 4; Rosmalen et al., 2012).

### Pleiotropic Effect Assessment

The pooled causal effects from the MR-Egger regression analysis are consistent with the IVW results: an estimated bate of T2DM per SD higher IL-18 plasma level was 0.122 (95% CI 0.003 – 0.221, *P* = 0.044); the intercept size was 0.011 (95% CI -0.004 to 0.026, *P* = 0.158), suggesting that all variants were valid. There is no alternative pathway leading to the disease, and the IVW was applied under no pleiotropic effect.

## Discussion

In this study, we conducted an MR analysis to explore the causal effect of IL-18 plasma levels on the risk of T2DM. The estimated causal impact resulting from the IVW method was 1.14 (95% CI 1.03 – 1.26, *P* = 0.0117). Additionally, the sensitivity analysis and the MR-Egger regression analysis also provided adequate evidence that the results were not due to heterogeneity or pleiotropic effects of any single variant.

A major innovative aspect of this study design is the introduction of the concept of IVs in the association analysis. In the causal inference of observational studies, no matter how good an epidemiological research design is and how accurate the measurements, we cannot eliminate the potential, unmeasurable confounding factors. The MR study design follows the Mendelian inheritance law of “random allocation of alleles to offspring.” If the genotype is associated with the disease through the phenotype, it can use genotypes as a variable to infer the association between phenotype and disease, as shown in Figure 1.

Given the results of this study, we can almost certainly conclude that the IL-18-associated T2DM risk is mainly due to the role of pro-inflammatory cytokines in β-cell dysfunction. Islet inflammations cause serious tissue lesions in both T1DM and T2DM. Upon infiltrating into the islet, the immune cells secrete a variety of pro-inflammatory cytokines, such as IL-1β, tumor necrosis factor alpha (TNF-α), and γ-interferon, and cause islet cell function defects and diabetes (Morgan et al., 2014; Marchetti, 2016; Eguchi and Nagai, 2017). The question of causality between T2DM and elevated IL-18 levels is answered in this study, as we have demonstrated that pro-inflammatory cytokines have a causal effect on T2DM. Our study not only aids in the development of prognostic techniques for diabetes and its complications but also provides a more comprehensive strategy for all types of clinical drug regimens to circumvent the risk of T2DM. Especially for non-T2DM treatments that can increase IL-18 expression, more stringent control, and careful handling are needed. For example, bacillus Calmette-Guérin (BCG) vaccines, which have been used for nearly a 100 years, have been confirmed in 2002 to cause a large increase in the expression of IL-1 family members, including IL-18, after vaccination (Lyons et al., 2002). Attention should be paid to the avoidance of virulence factors caused by the treatment process, and the rationality and safety of various medical treatments should be comprehensively evaluated.

Finding a solution for the high IL-18 levels may be a lengthy task. For instance, Schrezenmeir et al. used probiotic oligosaccharides to reduce the production of pro-inflammatory cytokines in intestinal cells and effectively reduced the burden of self-immunity (Zenhom et al., 2011). The use of statins can also effectively inhibit the expression of pro-inflammatory cytokines in CrFK cells infected with influenza A virus (Mehrbod et al., 2012). In cohort trials of patients with Alzheimer’s disease, researchers also found that ascorbic acid, α-tocopherol, and β-carotene can reduce oxidative stress and pro-inflammatory cytokine production in monocytes (de Oliveira et al., 2012). At the same time, daily exercise aids in reducing plasma levels of pro-inflammatory cytokines; early treadmill exercise reduced the production of pro-inflammatory factors in mice and even alleviated anxiety symptoms after cerebral ischemia (Zhang Q. et al., 2017).

Inevitably, this study has some minor limitations. When studying a single phenotypic variable, other phenotypes become confounding factors, so we introduced the MR concept, based on Mendel’s second law, the “law of independent assortment,” to solve this problem and to control genetic factors of different traits. We can insulate other pathway effects by linking genetic loci that control a single phenotype to the disease. But, as stated, the possibility of “non-isolation” still exists for some phenotypes that have not yet been completely described and which may be regulated by the same set of genetic loci. However, with the rapid updates and development of the databases, we expect this issue will be solved soon, e.g., by methods such as link prediction (Cheng et al., 2016, 2018a,b; Zeng et al., 2017; Jiang et al., 2018; Zhang et al., 2018; Ding et al., 2019) or artificial intelligence (Cabarle et al., 2017; Liu et al., 2017; Zhang X. et al., 2017; Cheng and Hu, 2018; Dao et al., 2018; Feng et al., 2018; Pan et al., 2018; Song et al., 2018; Tang et al., 2018; Wei et al., 2018; Xu et al., 2018a,b; Yang et al., 2018; Zhu et al., 2019). With the discovery of new IL-18 variants and the large collection of results of randomized controlled trials, we anticipate the discovery of more non-coding biomarkers for novel diagnostic or therapeutic strategies for T2DM (Zou et al., 2015; Lu et al., 2016; Liu et al., 2017; Wei et al., 2017a,b; Cheng et al., 2018c; Zeng et al., 2018).

## Author Contributions

HZ wrote the manuscript. JH and LC carried out the experimental method design. S-LL did the supervision and modification of the article.

## Conflict of Interest Statement

The authors declare that the research was conducted in the absence of any commercial or financial relationships that could be construed as a potential conflict of interest.

## Footnotes

1 http://diagram-consortium.org/about.html

2 http://www.cog-genomics.org/plink/1.9/ld#r

3 http://cran.r-project.org/web/packages/meta/index.html
